# The occurrence of early atrial fibrillation after cardiac valve operation and the establishment of a nomogram model

**DOI:** 10.3389/fcvm.2023.1036888

**Published:** 2023-04-17

**Authors:** Sailan Li, Haoruo Zhang, Xiaoqin Liao, Xin Yan, Liangwan Chen, Yanjuan Lin, Yanchun Peng

**Affiliations:** ^1^ Department of Cardiovascular Surgery, Fujian Medical University Union Hospital, Fuzhou, China; ^2^ Department of Clinical Medicine, Fujian Medical University, Fuzhou, China; ^3^ Department of Nursing, Fujian Medical University Union Hospital, Fuzhou, China

**Keywords:** postoperative atrial fibrillation, cardiac valve surgery, thyrotropin, nomogram model, risk factors

## Abstract

**Background:**

Postoperative atrial fibrillation (POAF) is a common complication after cardiac surgery, which is associated with age and massive bleeding. However, whether thyroid hormone (TH) level can affect POAF remains controversial.

**Aim:**

To investigate the occurrence and risk factors of POAF, in particular, the preoperative TH level of patients was introduced into this study as a variable for analysis, and a column graph prediction model of POAF was constructed.

**Methods:**

Patients who underwent valve surgery in Fujian Cardiac Medical Center from January 2019 to May 2022 were retrospectively analyzed and divided into POAF group and NO-POAF group. Baseline characteristics and relevant clinical data were collected from the two groups of patients. Independent risk factors for POAF were screened using univariate analysis and binary logistic regression analysis, and a column line graph prediction model was established based on the regression analysis results, and the diagnostic efficacy and calibration of the model were evaluated using the Receiver Operating Characteristic Curve (ROC) and calibration curve.

**Results:**

A total of 2,340 patients underwent valve surgery, excluding 1,751 patients, a total of 589 patients were included, including 89 patients in POAF group and 500 patients in NO-POAF group. The total incidence of POAF was 15.1%. The results of the Logistics regression analysis showed that gender, age, leukocytes and TSH were risk factors of POAF. The area under the ROC curve of the nomogram prediction model for POAF was 0.747 (95% CI: 0.688–0.806, *P *< 0.001), with a sensitivity of 74.2% and specificity of 68%. Hosmer-Lemeshow test showed *χ*^2 ^= 11.141, *P *= 0.194 > 0.05, the calibration curve was well fitted.

**Conclusion:**

The results of this study show that gender, age, leukocyte and TSH are risk factors of POAF, and the nomogram prediction model has a good prediction effect. Due to the limited sample size and included population, more studies are needed to validate this result.

## 1. Introduction

Postoperative atrial fibrillation (POAF) is a common complication after cardiac surgery, and 17.3%–76% of patients develop POAF after cardiac surgery (1–4). POAF usually occurs during hospitalization, has a short duration, recovers spontaneously without treatment, and rarely develops into chronic diseases (5, 6). However, POAF is significantly associated with increased mortality, length of hospital stay, and medical costs (7, 8). Therefore, the prevention of POAF after cardiac surgery is of great significance to promote disease recovery and reduce mortality (9, 10).

The relationship between thyroid hormone levels and disease has attracted more and more attention. Relevant datas show (11–13) that thyroid hormone changes within the normal range will also have an impact on the outcome of the disease, which may be related to impaired peripheral thyroxine deiodination and down-regulation of enzyme activity. Thyroid hormones play a wide range of roles in the cardiovascular system, causing arrhythmias, atherosclerotic vascular diseases, dyslipidemia and heart failure, which lead to an increased risk of death (14, 15).

Although previous studies have shown that the occurrence of POAF in patients undergoing heart valve surgery is correlated with age and hemorrhage (16, 17), however, no studies have investigated the effect of thyroid hormone levels on the occurrence of POAF in heart valves. Therefore, we introduced thyroid hormone levels into this study for analysis and constructed a nomogram prediction model in order to provide a more comprehensive understanding of the risk factors for the occurrence of POAF and to provide a reference for the prevention of POAF, so as to promote the life safety of patients.

## 2. Methods

### 2.1. Patients

This study collected patients who underwent valve surgery in Fujian Cardiac Medical Center from January 2019 to May 2022. All patients were ≥18 years old and had no preoperative atrial fibrillation, metabolic diseases, malignant tumors, history of autoimmune diseases, or severe hepatic or renal insufficiency. Exclusion criteria: patients with a history of heart disease, patients with thyroid disease.

### 2.2. Data collection

The baseline datas, preoperative, intraoperative and postoperative clinical datas of the patients were collected retrospectively. Baseline datas include gender, age, body mass index (BMI), smoking, alcohol consumption, hypertension and diabetes. Preoperative clinical datas include cardiac function classification (NYHA cardiac function classification divides cardiac function impairment into grades I to IV according to the activity of induceing heart failure symptoms), left ventricular ejection fraction, laboratory indicators. Intraoperative clinical datas include operation duration and cardiopulmonary bypass duration. Postoperative clinical datas include days of intensive care unit stay, duration of mechanical ventilation, and cardiac output (CO).

### 2.3. Assessment of atrial fibrillation

All patients after heart valve surgery were admitted to ICU for continuous vital signs monitoring, and whether the patient had atrial fibrillation was judged according to the data of the ECG monitoring instrument (18). POAF can be diagnosed if the patient has no AF before operation and is detected to have AF after operation. Atrial fibrillation that occurs from the day to 7 days after surgery is considered as early postoperative POAF.

### 2.4. Statistical analysis

SPSS24.0 software was used for datas analysis in this study. Continuous variables were expressed as mean ± standard deviation, and comparisons between groups were analyzed by two-independent sample *t*-test or Wilcoxon rank-sum test. Categorical variables were expressed as counts and percentages, and comparisons between groups were performed using *χ*^2^ test or Fisher's exact test. The independent risk factors of POAF were screened by binary Logistic regression analysis. Risk prediction models were developed, column line plots were drawn using R 4.2.2 software, and internal validation was performed. The area Under the Curve (AUC) of Receiver Operating Characteristic Curve (ROC) was used to evaluate the model differentiation, the Hosmer-Lemeshow goodness of fit test (H-L test) and calibration curve were used to evaluate the calibration degree of the model.

## 3. Results

### 3.1. Analysis of patients' inclusion

**Figure 1:**
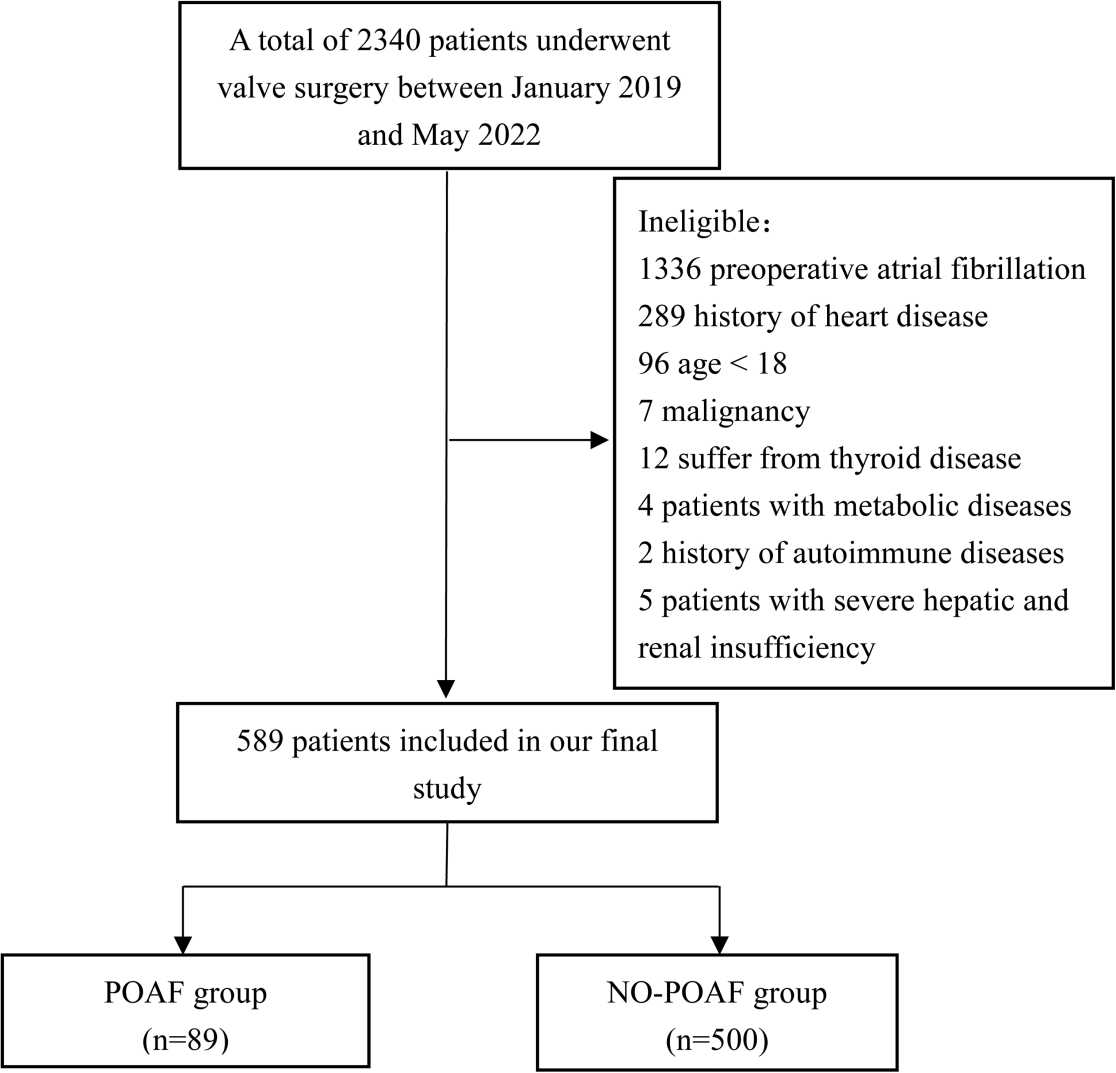
Patienst inclusion flow chart.

There are a total of 2,340 patients underwent valve surgery from January 2019 to May 2022. 1,751 patients were excluded (including 1,336 patients with preoperative atrial fibrillation, 289 patients with a history of heart diseases, 96 patients were younger than 18, 7 patients with malignant tumors, 12 patients with thyroid diseases, 4 patients with metabolic diseases, 2 patients with a history of autoimmune diseases, 5 patients with severe hepatic and renal insufficiency), a total of 589 patients were finally enrolled, there were 89 patients in POAF group and 500 patients in NO-POAF group. The flow chart of patients’ inclusion is shown in Figure 1.

### 3.2. Comparison of clinical datas between the two groups

#### 3.2.1. Baseline patients' characteristics

Table 1 shows the baseline characteristics of the two groups of patients. Among 589 patients, 89 (15.1%) were in the POAF group and 500 (84.9%) were in the NO-POAF group. Compared with the NO-POAF group, the age (61.067 ± 9.875 vs. 57.224 ± 11.517), the proportion of females (60.7% vs. 40%) and the proportion of hypertension patients (56.2% vs. 43.2%) in the POAF group were higher, and the differences were statistically significant (*P *< 0.05). There was no significant difference in BMI, smoking, drinking and diabetes between the two groups (*P *> 0.05). The difference is compared in Table 1.

**Table 1:** Baseline patients characteristics.

Variable	POAF group (n=89)	NO-POAF group (n=500)	Testing statistic	*P*-Value
Age	62.19 ± 10.20	57.18 ± 11.50	3.847	0.000
Gender			9.512	0.002
Male, *n* (%)	35 (39.3)	285 (57.0)		
Female *n* (%)	54 (60.7)	215 (43.0)		
BMI	23.20 ± 3.50	22.96 ± 3.78	0.557	0.577
Smoke			0.170	0.680
Yes *n* (%)	13 (14.6)	65 (13.0)		
No *n* (%)	76 (85.4)	435 (87.0)		
Brink			0.565	0.452
Yes *n* (%)	14 (15.7)	64 (12.8)		
No *n* (%)	75 (84.3)	436 (87.2)		
Hypertension			5.14	0.023
Yes *n* (%)	50 (56.2)	216 (43.2)		
No *n* (%)	39 (43.8)	284 (56.8)		
Biabetes			3.572	0.059
Yes *n* (%)	38 (42.7)	162 (32.4)		
No *n* (%)	51 (57.3)	338 (67.6)		

Note: BMI: Body mass index.

#### 3.2.2. Clinical datas of the patients

Table 2 Compares the clinical data of the two groups of patients. There were statistically significant differences in leukocyte, TSH and extracorporeal circulation time between the two groups (*P* < 0.05).

**Table 2:** Preoperative data of two groups of patients.

Variable	POAF group (n=89)	NO-POAF group (n=500)	Testing statistic	*P*-Value
Preoperative data comparison				
Pulmonary infection			1.479	0.477
Yes *n* (%)	13 (14.6)	97 (19.4)		
No *n* (%)	76 (85.4)	403 (80.6)		
NYHA			15.075	0.064
I	0 (0)	0 (0)		
II	58 (65.2)	382 (76.4)		
III	30 (33.7)	111 (22.2)		
IV	1 (1.1)	7 (1.4)		
LVEF (%)	60.54 ± 10.41	62.20 ± 11.31	0.959	0.338
Leukocyte 10^9^/L	13.30 ± 3.73	11.37 ± 3.65	4.570	0.000
Neutrophile Granulocyte 10^9^/L	10.24 ± 2.58	9.69 ± 2.63	1.832	0.067
Lymphocyte 10^9^/L	6.87 ± 1.39	6.67 ± 1.66	1.064	0.288
Monocyte 10^9^/L	0.53 ± 0.16	0.54 ± 0.16	0.799	0.425
Erythrocyte 10^12^/L	4.60 ± 0.57	4.58 ± 0.79	0.287	0.774
Platelet 10^9^/L	182.17 ± 58.19	186.54 ± 57.12	0.663	0.508
Hemoglobin g/L	131.39 ± 14.11	133.86 ± 13.94	1.537	0.125
TSH mIU/L			55.494	0.000
<5.45 mIU/L *n* (%)	65 (73.0)	479 (95.8)		
≥5.45 mIU/L *n* (%)	24 (27.0)	21 (4.2)		
FT3 pmol/L			0.439	0.508
<22.82 pmol/L *n* (%)	84 (94.4)	462 (92.4)		
≥22.82 pmol/L *n* (%)	5 (5.6)	38 (7.6)		
FT4 pmol/L			-	0.561
<5.81 pmol/L *n* (%)	88 (98.9)	496 (99.2)		
≥5.81 pmol/L *n* (%)	1 (1.1)	4 (0.8)		
Intraoperative data comparison				
Operation time(min)	270.82 ± 63.36	258.17 ± 66.47	1.665	0.096
Cardiopulmonary bypass time(min)	123.58 ± 31.31	117.30 ± 30.17	2.214	0.027
Postoperative data comparison				
ICU stay (d)	2.90 ± 0.93	2.70 ± 1.18	1.508	0.077
Mechanical ventilation time (h)	19.10 ± 5.75	18.70 ± 6.11	0.581	0.561
CO L/min	6.09 ± 3.03	7.098 ± 5.84	1.202	0.230

Note: LVEF, left ventricular ejection fraction; TSH, thyroid stimulating hormone; FT3, free triiodothyronine; FT4, free tetraiodothyronine.

### 3.3. Binary logistic regression analysis

In Table 3, binary logistic regression analysis was performed with POAF as the dependent variable and gender, age, hypertension, leukocytes, TSH, and duration of extracorporeal circulation as independent variables. The results showed that gender (OR: 1.903, 95% CI: 1.140–3.176, *P *= 0.014), age (OR: 1.040, 95% CI: 1.015–1.006, *P *= 0.002), leukocytes (OR: 1.144, 95% CI: 1.072–1.221, *P *= 0.000), TSH (OR. 8.910, 95% CI: 4.396–18.057, *P *= 0.000) and other variables were risk factors for POAF in patients undergoing heart valve surgery.

**Table 3:** Binary logistic regression analysis.

Variable	*B*	SE	Wald value	*P*	OR	95% CI	
						Lower	Upper
Gender	0.643	0.261	6.061	0.014	1.903	1.140	3.176
Age	0.039	0.012	9.940	0.002	1.040	1.015	1.006
Hypertension	−0.433	0.256	2.861	0.091	0.649	0.393	1.071
Leukocyte 10^9^/L	0.135	0.033	16.612	0.000	1.144	1.072	1.221
TSH mIU/L	2.187	0.360	36.830	0.000	8.910	4.396	18.057
Cardiopulmonary bypass time (min)	0.004	0.004	0.997	0.318	1.004	0.996	1.013
Constant	−6.628	1.025	41.783	0.000	0.001		

### 3.4. POAF risk prediction line graph model

#### 3.4.1. Constructing a line graph prediction model

**Figure 2:**
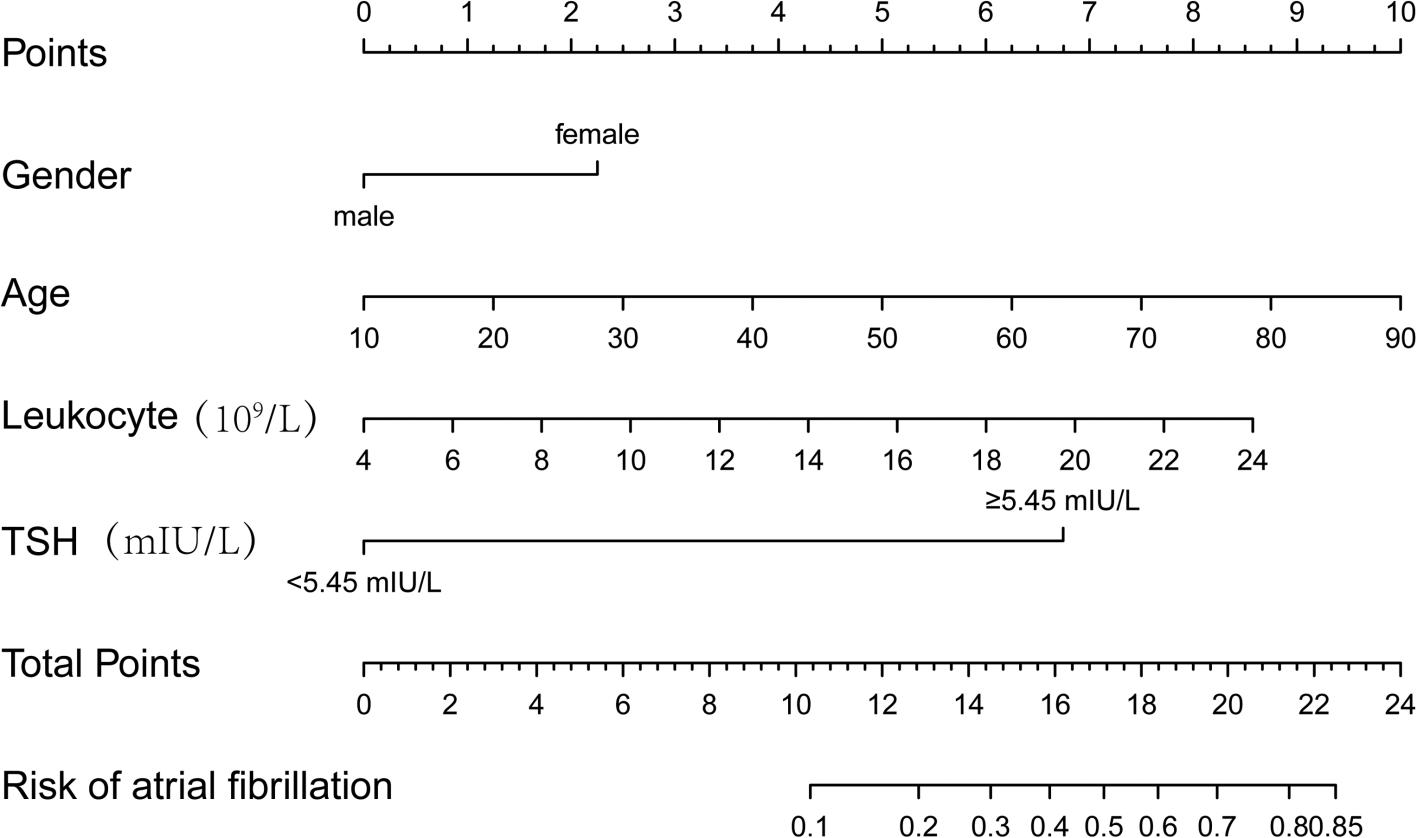
POAF risk prediction line graph model.

Columnar line graph of POAF risk prediction was drawn based on risk factors identified by binary logistic regression analysis. The column line plot prediction model showed that an increase of 2.25 points for women; 1.25 points for each 10-year increase in age; 0.75 points for each 2 units increase in leukocytes; and 6.75 points for TSH ≥ 5.45 mIU/L. The scores of the above risk factors were summed to calculate the total score, and the probability of occurrence of POAF corresponding to the column line graph was calculated based on the total score (Figure 2).

#### 3.4.2. Distinguishability of the model

**Figure 3:**
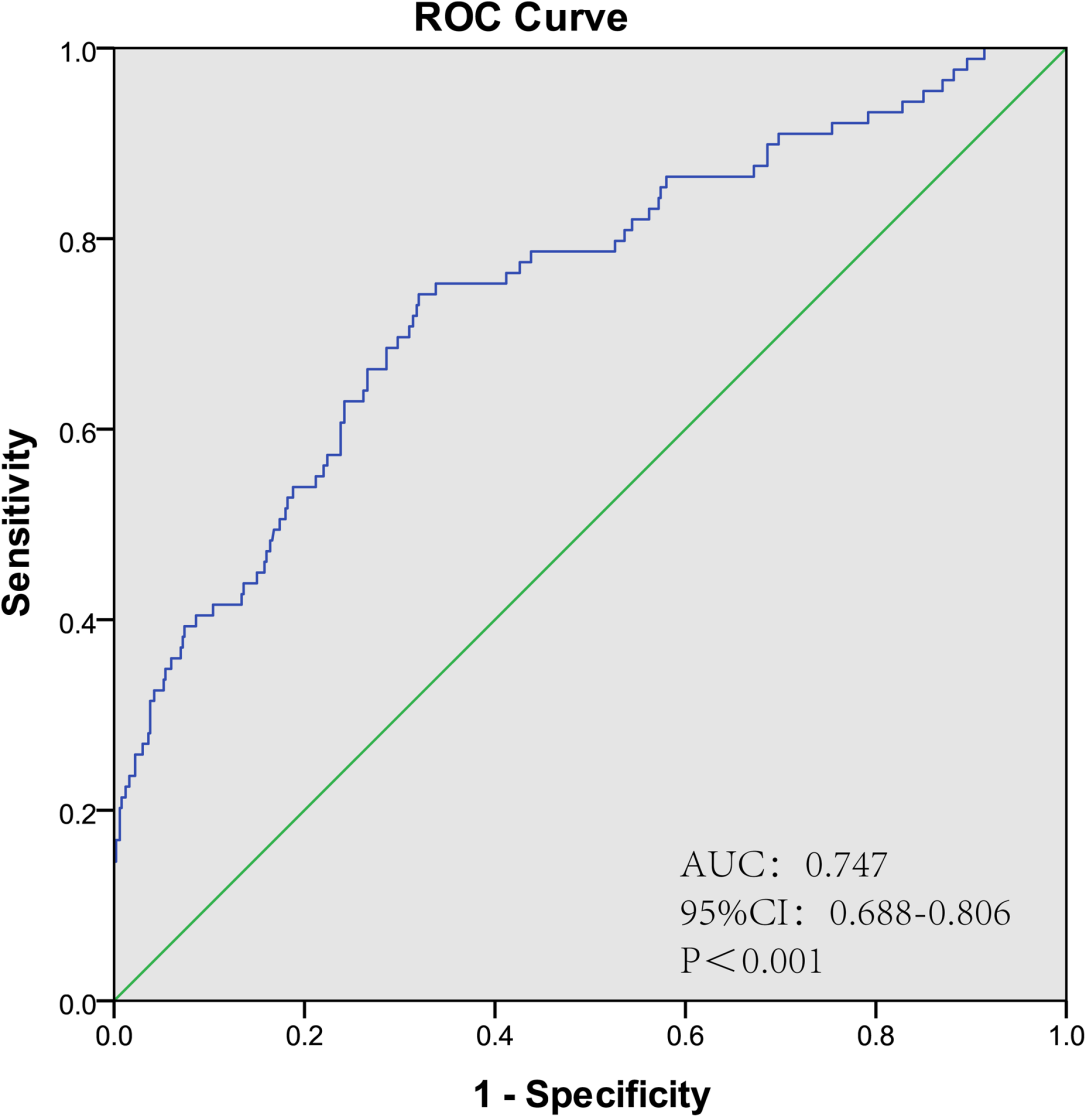
ROC curve of POAF risk prediction model.

The ROC curve was plotted according to the prediction model, and the results showed an AUC of 0.747 (95% CI: 0.688–0.806, *P *< 0.001), a sensitivity of 74.2% and a specificity of 68%, suggesting that the model has a good discrimination. The ROC curve of POAF prediction model is shown in Figure 3.

#### 3.4.3. Calibration degree of the model

**Figure 4:**
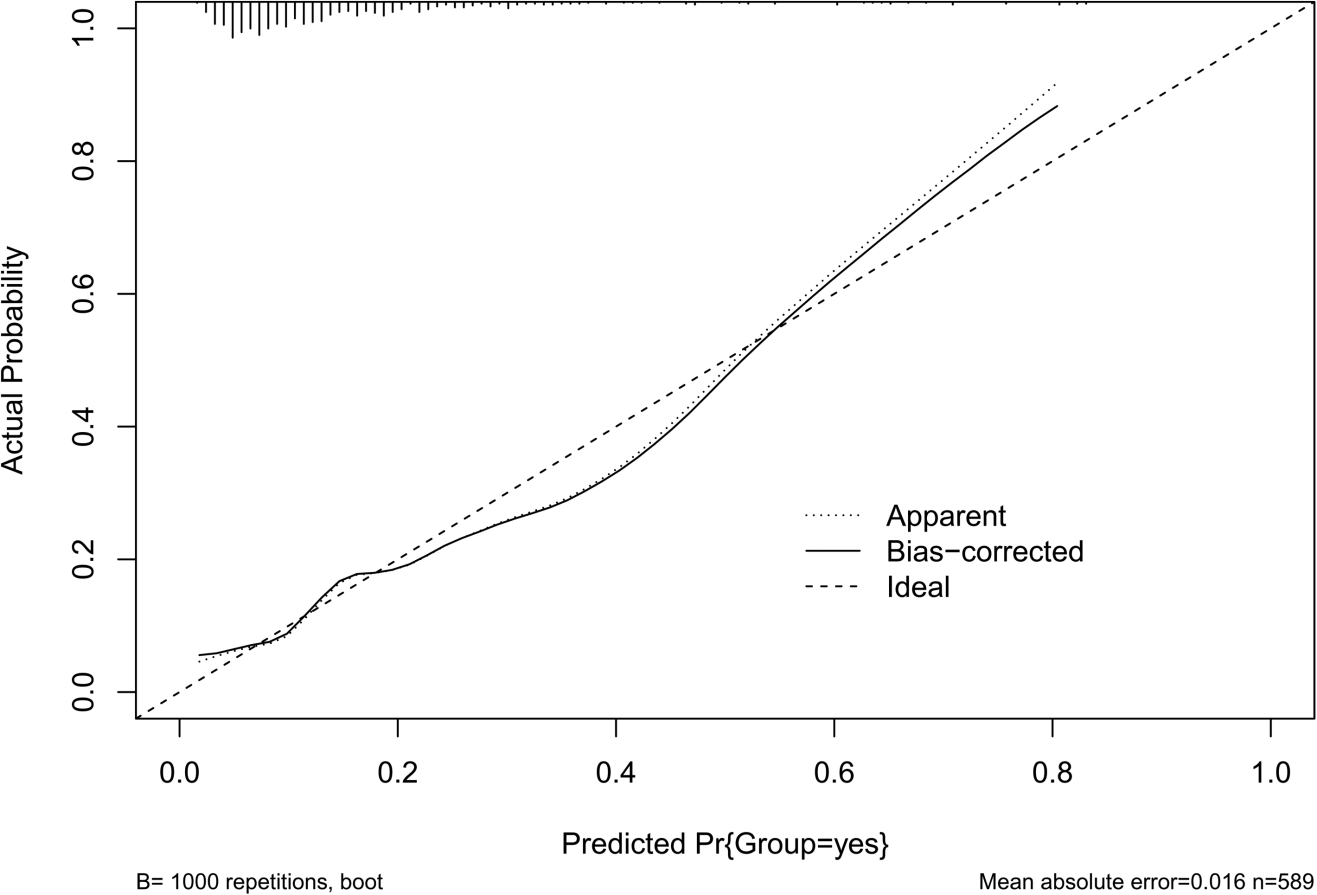
The calibration curve of POAF risk prediction model.

The results of the H-L test showed *χ*^2 ^= 11.141, *P *= 0.194 > 0.05, and the calibration curve was well fitted as shown by bootstrap method with 1,000 repetitions (Figure 4), indicating that our predicted and observed values are close and in good agreement.

## 4. Discussion

### 4.1. Risk factors for POAF

There's a high incidence of POAF after cardiac surgery. Our study showed that the incidence of POAF in patients undergoing heart valve surgery was 15.1%, which was consistent with previous research results (19–21). Binary logistic regression analysis showed that gender, age, leukocyte and TSH were strongly correlated with the occurrence of POAF.

The results of this study showed that the age of patients in the POAF group was higher than that in the NO-POAF group, and the ROC curve showed that the optimal cut-off value of age was 64.5. Recent studies have also shown (22, 23) that advanced age is a risk factor for POAF in patients after cardiac surgery, which is consistent with the results of this study. It may be related to the frailty and loss of ventricular compliance in older patients (24, 25). Early identification of advanced age as a risk factor for POAF can enable medical staff to take targeted preventive measures before operation and carry out effective perioperative management.

Not only age is a risk factor for POAF, but gender can also have an important impact on the occurrence of POAF. In our study, women were a predictor of POAF in postoperative cardiac patients, and there was a strong correlation between women and the occurrence of POAF, which is consistent with the study of Li et al. (26). The study of Cheng et al. (27) also confirmed a higher recurrence rate of AF in women. This may be related to faster atrial remodeling and a higher rate of arrhythmia recurrence in women (28). Overall, it is not clear why gender difference has an impact on the recurrence of AF, and there are few relevant studies, which need to be further investigated.

Leukocyte is a risk factor for POAF. This may be related to increased local inflammation and pericardial effusion around the heart as a result of cardiac surgery. Postoperative pericardial effusion is highly oxidized, the myocardium itself produces pro-inflammatory cytokines, and local inflammation can directly affect cardiac function (29, 30). Inflammation in the pericardial cavity may alter myocardial cell electrical activity and apoptosis, causing elevated leukocyte counts, which can lead to action potential heterogeneity and arrhythmia formation and propagation (31, 32). Therefore, prevention of inflammation may be able to reduce the incidence of POAF (33).

In addition, our results showed a significant effect of TSH levels on the occurrence of POAF. The studies by Morishima et al., Sairaku et al. (34, 35) support our findings, while Baumgartner et al. (36) concluded that there was no significant correlation between TSH levels and AF. Considering the existence of different populations included in different studies differences, future studies with larger samples and multicenter studies are needed to fully analyze the effects of TSH levels on different populations.

### 4.2. POAF risk prediction line graph model

In this study, a POAF risk column line graph prediction model was established, especially introducing TH as a variable for analysis in this study. The risk prediction model developed in this study had good discrimination and calibration. Compared with previous studies that analyzed the influencing factors of AF (37, 38), the column line graph prediction model we developed was able to score patients more intuitively and quickly to predict the probability of POAF.

## 5. Limitations of the study

There are some limitations to our study. First of all, more large-sample, multicenter studies are needed to further evaluate the effect of TSH level on POAF due to the relatively small number of cases and single population included in the study. Secondly, due to the limitation of sample size, we only compared the difference of TSH level between the POAF group and the NO-POAF group, and did not stratify the TSH level. Third, this study was a retrospective study, which only evaluated early POAF and did not analyze the long-term occurrence of POAF.

## 6. Conclusion

We investigated the risk factors of POAF after heart valve surgery, and the prediction model constructed by the column graph has good predictive ability. However, due to the influence of sample size and included groups, more large-sample and multi-center studies are needed to verify the results of this study.

## Data Availability

The raw data supporting the conclusions of this article will be made available by the authors, without undue reservation.
